# Supplementary figures and images for: The effects of selected biologics and a small molecule on Health-Related Quality of Life in adult plaque psoriasis patients: A systematic review and meta-analysis
Source: PLoS One. 2020 Dec 3;15(12):e0241604. doi: 10.1371/journal.pone.0241604 (PMC7714099; doi:10.1371/journal.pone.0241604)

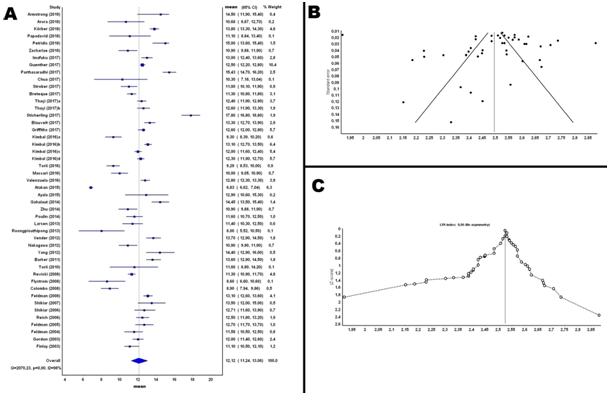

Supplement: S1 Fig — (TIF) [file pone.0241604.s003.tif]

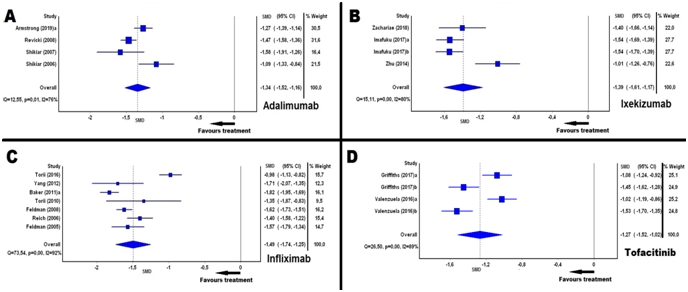

Supplement: S2 Fig — (TIF) [file pone.0241604.s004.tif]

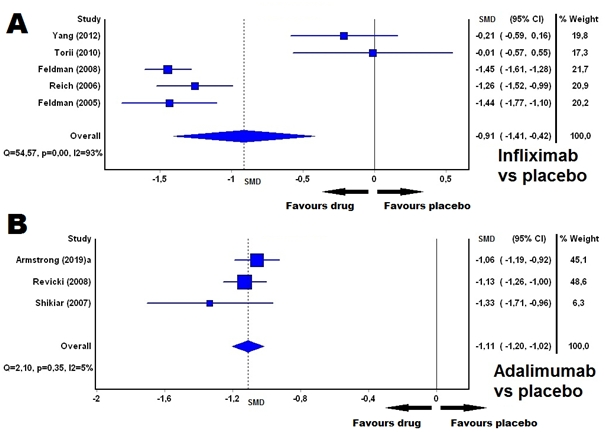

Supplement: S3 Fig — (TIF) [file pone.0241604.s005.tif]
